# Endothelial function is preserved in light to moderate alcohol drinkers but is impaired in heavy drinkers in women: Flow-mediated Dilation Japan (FMD-J) study

**DOI:** 10.1371/journal.pone.0243216

**Published:** 2020-12-03

**Authors:** Nozomu Oda, Masato Kajikawa, Tatsuya Maruhashi, Shinji Kishimoto, Farina Mohamad Yusoff, Chikara Goto, Ayumu Nakashima, Hirofumi Tomiyama, Bonpei Takase, Akira Yamashina, Yukihito Higashi

**Affiliations:** 1 Department of Cardiology, Hiroshima Prefectural Hospital, Hiroshima, Japan; 2 Division of Regeneration and Medicine, Medical Center for Translational and Clinical Research, Hiroshima University Hospital, Hiroshima, Japan; 3 Department of Cardiovascular Regeneration and Medicine, Research Institute for Radiation Biology and Medicine, Hiroshima University, Hiroshima, Japan; 4 Department of Physical Therapy, Hiroshima International University, Hiroshima, Japan; 5 Department of Stem Cell Biology and Medicine, Hiroshima University Graduate School of Biomedical Sciences, Hiroshima, Japan; 6 Department of Cardiology, Tokyo Medical University, Tokyo, Japan; 7 Division of Biomedical Engineering, National Defense Medical College Research Institute, Tokorozawa, Japan; University of Thessaly, GREECE

## Abstract

Light to moderate alcohol consumption has protective effects on all-cause death and coronary artery disease in women. It is thought that light to moderate alcohol consumption has a beneficial effect on vascular function in women. We measured flow-mediated vasodilation (FMD) in 702 women aged 17–86 years who provided information on alcohol consumption. We divided the subjects into four groups: non-drinkers (0 g/week), light drinkers (>0 to 140 g/week), moderate drinkers (>140 to 280 g/week) and heavy drinkers (>280 g/week). There was no significant difference in FMD among the four groups. Multivariate regression analysis revealed that alcohol consumption in non-drinkers and light drinkers was not an independent predictor of FMD (β = −0.001, P = 0.98). We compared 50 moderate drinkers and 50 non-drinkers matched for age and medical histories and 22 heavy drinkers and 22 non-drinkers in matched pair analysis. There was no significant difference in FMD between moderate drinkers and non-drinkers (8.2±4.3% vs. 8.1±3.5, P = 0.91), while FMD in heavy drinkers was significantly lower than that in non-drinkers (5.9±2.5% vs. 8.9±3.5%, P = 0.002). These findings suggest that heavy alcohol consumption is associated with endothelial dysfunction but that light to moderate alcohol consumption is not associated with endothelial dysfunction in women.

**Clinical trial registration information** This study was approved by principal authorities and ethical issues in Japan (University Hospital Medical Information Network UMIN000012952, 01/12/2009). www.umin.ac.jp/.

## Introduction

Humankind has enjoyed drinking alcohol from prehistory. According to some of the oldest records in history, ancient Egyptians were addicted to alcohol, and Cleopatra was also a drinker [1]. The drinking style of women has kept in step with the times. It is well known that there is a gender difference in drinking behavior. Women generally drink less alcohol than men do [2]. However, the opportunities for women to drink alcohol have been increasing in accordance with the changing social status of women in developed countries [3].

It has become more clinically important to target women as well as men for reduction in alcohol-related diseases including cardiovascular diseases. The effects of alcohol may be different in men and women. Several epidemiological studies have shown J-shape relations between alcohol consumption and all-cause mortality and cardiovascular events in women [4–6]. Alcohol drinking similarly has effects on the lipid profile [7–9], glycometabolism [10, 11], and coagulation fibrinolysis system [12–14] in both sexes.

Endothelial dysfunction is known to be the initial step in the pathogenesis of systemic atherosclerosis and plays an important role in the development of atherosclerosis [15, 16]. Flow-mediated vasodilation (FMD), an index of endothelium-dependent vasodilation, has been used for evaluation of endothelial function [17–19]. Several investigators including us have shown that endothelial dysfunction is an independent predictor of cardiovascular events [20–23]. In a previous study, we showed that even light to moderate alcohol consumption impaired endothelial function in men [24]. However, we speculated that light to moderate alcohol consumption has beneficial effects on endothelial function in women. There is little information on the relationship between alcohol consumption and endothelial function in women. Therefore, we evaluated the relationship between alcohol consumption and endothelial function assessed by FMD in women.

## Materials and methods

### Subjects

A total of 5314 Japanese (4107 men and 1207 women) aged 17 to 86 years who underwent health-screening examinations with agreement for examination of vascular function were registered in the Flow-mediated Dilation Japan Registry between April 1, 2010 and August 31, 2012 at 3 general hospitals in Japan. All Japanese employees have an obligation to undergo health screening every year under the regulations of the society-managed health insurance union. From the registry, 702 women aged 17 to 84 years who provided information on alcohol consumption (kind of beverage consumed and the frequency and amount of drinking) at health-screening examinations were recruited for this study (S1 Fig). Subjects with severe chronic heart failure (New York Heart Association level of more than III), severe valvular heart disease, arrhythmia that required treatment, or malignancy and patients being treated with steroids, nonsteroidal anti-inflammatory drugs or immunosuppressive drugs were excluded from the study. Alcoholic subjects with severe liver dysfunction or anemia caused by alcohol drinking were also excluded from the study. Hypertension was defined as systolic blood pressure of more than 140 mm Hg or diastolic blood pressure of more than 90 mm Hg, in a sitting position, on at least different three occasions. Diabetes was defined according to the American Diabetes Association [25]. Dyslipidemia was defined according to the third report of the National Cholesterol Education Program [26]. We defined smokers as those who were currently smoking. Hyperuricemia was defined as serum uric acid concentration of more than 7.0 mg/dL [27]. Framingham risk score (FRS) was calculated by points of risk factors: age, total cholesterol concentration, high-density lipoprotein (HDL) cholesterol concentration, systolic blood pressure, and smoking status [28]. The ethical committees of Hiroshima University Graduate School of Medicine approved the study protocol. This study was registered in the University hospital medical information network (UMIN000012952, 01/12/2009). The study was executed in accordance with the Good Clinical Practice guidelines. Written informed consent for participation in the study was obtained from all subjects. The need for parental consent was waived by the ethics committee. There were no subjects with written informed consent for participation in the study provided by proxies.

### Study protocol

A total of 702 women answered a questionnaire about alcohol intake for at least the past year, including questions on kind of beverage consumed, frequency of drinking in a week and daily drinking quantity (S1 File). Subjects who did not drink more than once a week were regarded as non-drinkers. There were no binge drinkers. We calculated the quantity of one-week absolute alcohol consumption. At first, the subjects were divided into a non-drinker group and a drinker group. Then the subjects were divided into 4 groups by alcohol consumption: non-drinker group (0 g/week), light drinker group (>0 to 140 g/week), moderate drinker group (>140 to 280 g/week), and heavy drinker group (>280 g/week). We compared 227 light drinkers with 227 non-drinkers matched for age and medical histories (hypertension, dyslipidemia and diabetes mellitus). Furthermore, we compared 50 moderate drinkers with 50 non-drinkers and 22 heavy drinkers with 22 non-drinkers matched for the same variables. We also conducted the same analysis as that described above in 372 premenopausal women who were not in the menstrual phase (176 non-drinkers, 149 light drinkers, and 35 moderate drinkers). Subsequently, we divided 172 postmenopausal women into 4 groups in accordance with alcohol consumption to remove the effects of the menstrual cycle. Postmenopausal women were defined as women aged over 40 years who answered that they experienced 12 consecutive months without menstruation. Finally, we evaluated the associations between alcohol consumption and FMD in 644 subjects who were not receiving drugs for hypertension, dyslipidemia, and diabetes mellitus (358 non-drinkers, 218 light drinkers, 48 moderate drinkers, and 20 heavy drinkers).

The protocol for this study has been described previously [24]. In brief, we measured brachial arterial responses to hyperemia in all participants. Subjects fasted overnight for at least 12 hours and abstained from caffeine, alcohol, smoking, and antioxidant vitamins on the day of the FMD examination. Measurement of FMD was performed with medication. The study began at 8:30 a.m. The participants were kept in a supine position in a quiet, dark and air-conditioned room (constant temperature of 22–25°C) throughout the study. After being in the supine position for 30 minutes, blood samples were obtained for measurement of basal fasting serum concentrations of total cholesterol, triglycerides, HDL cholesterol, low-density lipoprotein (LDL) cholesterol, gamma glutamyl transpeptidase (γ-GTP), creatinine, uric acid, glucose, and hemoglobin A1c (HbA1c).

### Calculation of the quantity of absolute alcohol consumption

We calculated the quantity of one-week absolute alcohol intake from the type of beverage, frequency of drinking in a week and quantity of one-time drinking. We estimated alcohol concentrations as follows: 5% for beer, 14% for wine, 15% for sake, 25% for shochu (Japanese vodka) and 43% for whiskey. The specific gravity of 1 mL of 1% alcohol was assumed to be 0.8 g. Absolute alcohol was calculated by the following formula: Absolute alcohol (g) = Alcohol drinking (mL) × Alcohol concentration (%)/100 × 0.8 (g/mL).

### Measurement of FMD

FMD was measured by ultrasonography with an automated edge tracking system (UNEX 18G, UNEX Co. Nagoya, Japan) as previously described [29]. The vascular response to 5-min reactive hyperemia in the brachial artery was used for assessment of endothelium-dependent FMD. The observers were blind to the form of examination.

### Laboratory measurements

Fasting blood samples for laboratory measurements were collected from the subjects before FMD measurement. Levels of serum total cholesterol, triglycerides, HDL cholesterol, γ-GTP, creatinine, uric acid, HbA1c, and plasma glucose were enzymatically measured at each participating institution. LDL cholesterol was calculated by the Friedewald formula. Estimated glomerular filtration rate (eGFR) was calculated by the following equation: 194 × serum creatinine^-1.094^ × age^-0.287^ (×0.739 if women).

### Statistical analysis

Continuous variables are presented as means ± standard deviation. Categorical variables are presented as percentages. All reported P values were 2-sided, and a P value <0.05 was considered statistically significant. Comparison of continuous variables between two groups was performed by using Student’s unpaired t-test and comparison among four groups was performed by using one-way analysis of variance. Comparison of categorical variables among groups was performed by the chi-squared test. Tukey’s post-hoc test was used to compare the differences in FMD between groups. We derived the relationships between FMD, alcohol consumption, and variables using Spearman’s correlation coefficients. The associations between alcohol consumption and endothelial function were evaluated by using a propensity score-matched population. A logistic regression model was used to estimate the propensity of alcohol intake categories based on variables associated with alcohol consumption, including age and prevalence of hypertension, hyperlipidemia, and diabetes. With these propensity scores using a caliper width of 0.2 standard deviations of the logit of the propensity score, two well-matched groups based on clinical characteristics were created for comparison of FMD values. The data were processed using Stata version 9 (Stata Co. College Station, Texas, USA) by a blinded statistician.

## Results

### Clinical characteristics

The baseline characteristics of the 702 subjects are summarized in Table 1. Of the 702 participants, 94 (13.4%) had hypertension, 184 (26.3%) had dyslipidemia, 19 (2.7%) had diabetes mellitus, 10 (1.4%) had hyperuricemia, and 8 (1.1%) were current smokers. The mean value of FMD was 7.3±3.7%.

**Table 1:** Clinical characteristics of the subjects.

Variables	Total (n = 702)	Alcohol consumption		P value
		Non-drinker (n = 390)	Drinker (n = 312)	
Age, years	45±14	47±14	42±13	<0.001
Body mass index, kg/m^2^	21.4±3.4	21.8±3.6	20.9±3.1	<0.001
Systolic blood pressure, mm Hg	118±18	121±18	114±17	<0.001
Diastolic blood pressure, mm Hg	73±12	75±12	71±12	<0.001
Heart rate, bpm	65±10	66±10	63±10	0.001
Total cholesterol, mg/dL	197±35	200±37	194±31	0.02
Triglycerides, mg/dL	79±48	84±51	71±44	0.001
HDL cholesterol, mg/dL	70±15	67±14	74±16	<0.001
LDL cholesterol, mg/dL	114±30	119±32	108±26	<0.001
γ-GTP, mg/dL	22±22	21±18	24±26	0.04
eGFR, mL/min/1.73m^2^	83.5±16.2	82.3±15.5	84.9±16.9	0.04
Uric acid, mg/dL	4.3±0.9	4.2±0.9	4.4±1.0	<0.001
Glucose, mg/dL	91±14	92±15	91±11	0.24
Hemoglobin A1c, %	5.3±0.8	5.4±0.7	5.2±0.9	<0.001
Framingham risk score, %	3.0±3.3	3.5±3.7	2.3±2.6	<0.001
Medical history, n (%)				
Hypertension	94 (13.4)	56 (14.4)	38 (12.2)	0.39
Dyslipidemia	184 (26.3)	118 (30.3)	66 (21.2)	0.006
Diabetes mellitus	19 (2.7)	11 (2.8)	8 (2.6)	0.83
Hyperuricemia	10 (1.4)	4 (1.0)	6 (1.9)	0.32
Current smoker, n (%)	8 (1.1)	1 (0.3)	7 (2.2)	0.01
Medication, n (%)				
RAS inhibitors	16 (2.3)	6 (1.5)	10 (3.2)	0.16
Beta-blockers	1 (0.1)	0 (0)	1 (0.3)	0.21
Calcium channel blockers	26 (3.7)	10 (2.6)	16 (5.1)	0.91
Statins	17 (2.4)	6 (1.5)	11 (3.5)	0.15
Antidiabetic drugs	6 (0.9)	4 (1.0)	2 (0.6)	0.25
Insulin	0 (0)	0 (0)	0 (0)	N/A
Flow-mediated vasodilation, %	7.3±3.7	7.3±3.8	7.2±3.6	0.96

HDL indicates high-density lipoprotein; LDL, low-density lipoprotein; γ-GTP, gamma glutamyl transpeptidase; eGFR, estimated glomerular filtration rate; RAS, renin-angiotensin system; and N/A, not available.

### FMD in the non-drinker group and drinker group

Clinical characteristics of the subjects in the non-drinker group and the drinker group are summarized in Table 1. There were significant differences in age, body mass index (BMI), systolic blood pressure, diastolic blood pressure, heart rate, total cholesterol, triglycerides, HDL cholesterol, LDL cholesterol, γ-GTP, eGFR, uric acid, HbA1c, FRS, prevalence of dyslipidemia, and percentage of current smokers between the non-drinker group and the drinker group. Other parameters were not significantly different between the two groups. The mean values of FMD in the non-drinker group and drinker group were 7.3±3.8% and 7.2±3.6%, respectively. There was no significant difference in FMD between the two groups (P = 0.96).

### Relationships between FMD, alcohol consumption and variables

Table 2 shows univariate relations between FMD, alcohol consumption and variables. FMD correlated significantly with age (ρ = −0.41, P<0.01), BMI (ρ = −0.15, P<0.01), systolic blood pressure (ρ = −0.18, P<0.01), diastolic blood pressure (ρ = −0.12, P<0.01), total cholesterol (ρ = −0.24, P<0.01), triglycerides (ρ = −0.21, P<0.01), LDL cholesterol (ρ = −0.24, P<0.01), eGFR (ρ = 0.21, P<0.01), uric acid (ρ = −0.08, P<0.05), glucose (ρ = −0.13, P<0.01), HbA1c (ρ = −0.24, P<0.01) and FRS (ρ = −0.38, P<0.01). Other parameters were not correlated with FMD.

**Table 2:** Univariate analysis of relationship between FMD, alcohol consumption and variables.

Variables	FMD	Alcohol consumption
Age, years	−0.41†	−0.16†
Body mass index, kg/m^2^	−0.15†	−0.14†
Systolic blood pressure, mm Hg	−0.18†	−0.19†
Diastolic blood pressure, mm Hg	−0.12†	−0.21†
Heart rate, bpm	0.05	−0.12†
Total cholesterol, mg/dL	−0.24†	−0.08*
Triglycerides, mg/dL	−0.21†	−0.16†
HDL cholesterol, mg/dL	0.02	0.23†
LDL cholesterol, mg/dL	−0.24†	−0.19†
γ-GTP, mg/dL	−0.07	0.19†
eGFR, mL/min/1.73m^2^	0.21†	0.08*
Uric acid, mg/dL	−0.08*	0.15†
Glucose, mg/dL	−0.13†	−0.04
Hemoglobin A1c, %	−0.24†	−0.20†
Framingham risk score, %	−0.38†	−0.20†
Smoking	−0.02	0.10†
Alcohol consumption, g/week	0.0003	
Flow-mediated vasodilation, %		0.0003

FMD indicates flow-mediated vasodilation; FRS, Framingham risk score; HDL, high-density lipoprotein; LDL, low-density lipoprotein; γ-GTP, gamma glutamyl transpeptidase; and eGFR, estimated glomerular filtration rate.

Univariate analysis of the relations between FMD, alcohol consumption and variables (Spearman’s rank coefficients analysis) *P<0.05, †P<0.01

Alcohol consumption had significant correlations with age (ρ = −0.16, P<0.01), BMI (ρ = −0.14, P<0.01), systolic blood pressure (ρ = −0.19, P<0.01), diastolic blood pressure (ρ = −0.21, P<0.01), heart rate (ρ = −0.12, P<0.01), total cholesterol (ρ = −0.08, P<0.05), HDL cholesterol (ρ = 0.23, P<0.01), LDL cholesterol (ρ = −0.19, P<0.01), triglycerides (ρ = −0.16, P<0.01), γ-GTP (ρ = 0.19, P<0.01), eGFR (ρ = 0.08, P<0.05), uric acid (ρ = 0.15, P<0.01), HbA1c (ρ = −0.20, P<0.01), FRS (ρ = −0.20, P<0.01) and smoking (ρ = 0.10, P<0.01). Other parameters were not correlated with alcohol consumption.

### Relationship between FMD and alcohol consumption

There was no significant difference in FMD between the non-drinker group and drinker group (Table 1). FMD was not correlated with alcohol consumption in univariate analysis (ρ = 0.0003, P = 0.99) (Table 2).

**Fig 1:**
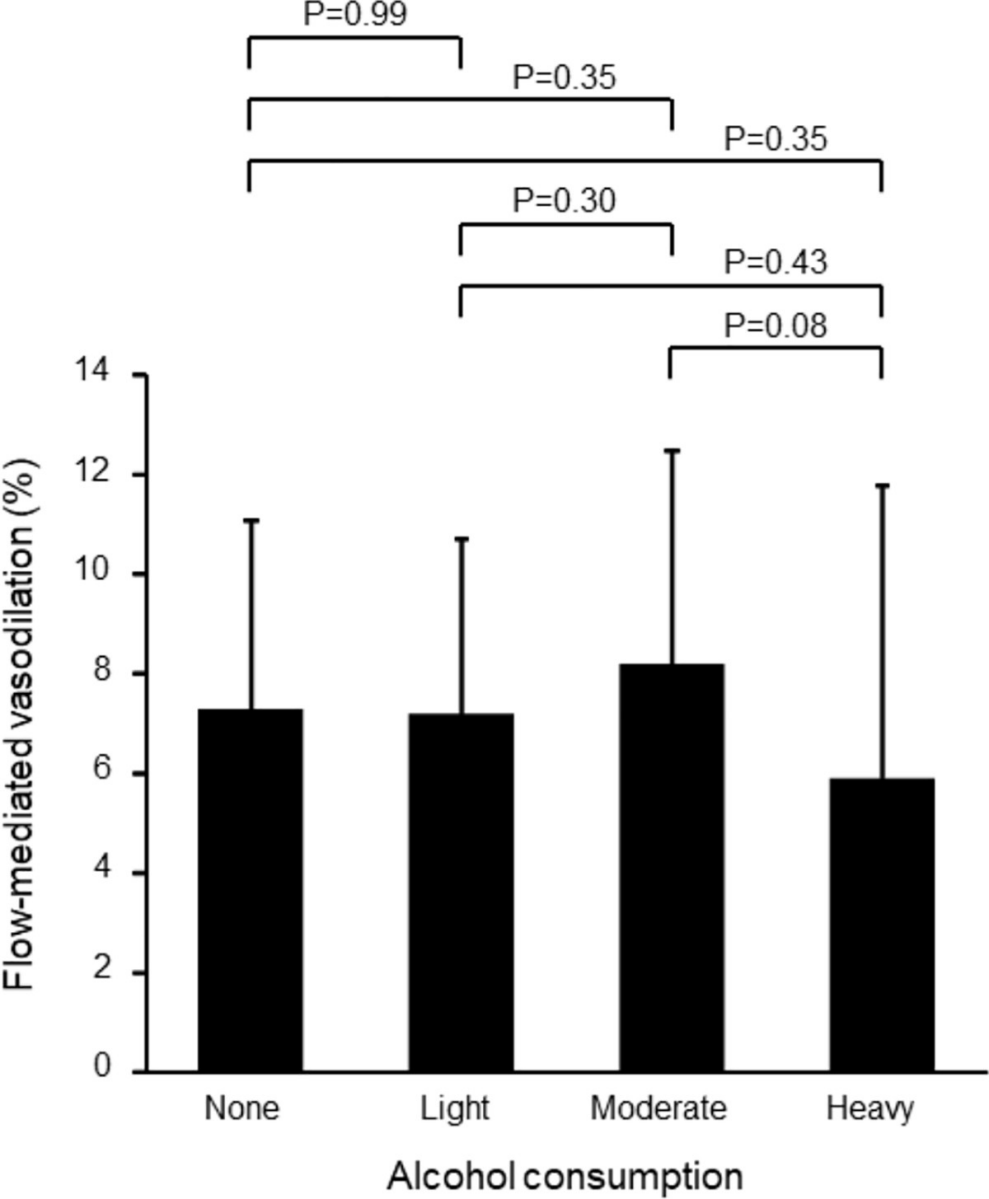
Bar graphs show flow-mediated vasodilation in subjects classified into four groups based on alcohol consumption.

The subjects were divided into four groups (non-drinker group and three drinker groups) to evaluate the difference in FMD in accordance with the amount of alcohol intake. Clinical characteristics of the subjects in the four groups are summarized in Table 3. There were significant differences in age, BMI, systolic blood pressure, diastolic blood pressure, heart rate, triglycerides, HDL cholesterol, LDL cholesterol, uric acid, HbA1c and FRS among the four groups. Other parameters were not significantly different among the four groups. Fig 1 shows the values of FMD in the four groups. The values of FMD were 7.3±3.8% in the non-drinker group, 7.2±3.5% in the light drinker group, 8.2±4.3% in the moderate drinker group and 5.9±2.5% in the heavy drinker group. There were no significant differences in FMD among the four groups (P = 0.11).

**Table 3:** Clinical characteristics of the subjects in accordance with alcohol consumption.

Variables	Alcohol consumption				P value for trend
	None 0 g/week (n = 390)	Light 0< to 140 g/week (n = 240)	Moderate 140< to 280 g/week (n = 50)	Heavy >280 g/week (n = 22)	
Age, years	47±14	43±13	39±13	42±17	<0.001
Body mass index, kg/m^2^	21.8±3.6	21.1±3.3	20.1±2.1	20.8±2.6	0.002
Systolic blood pressure, mm Hg	121±18	115±17	108±14	117±24	<0.001
Diastolic blood pressure, mmHg	75±12	72±13	68±9	71±12	<0.001
Heart rate, bpm	66±10	63±8	64±14	68±11	<0.001
Total cholesterol, mg/dL	200±37	195±33	193±28	193±27	0.15
Triglycerides, mg/dL	84±51	73±44	67±38	75±52	0.009
HDL cholesterol, mg/dL	67±14	72±15	78±16	78±17	<0.001
LDL cholesterol, mg/dL	119±32	110±27	102±24	100±25	<0.001
γ-GTP, mg/dL	21±18	23±25	28±33	30±22	0.052
eGFR, mL/min/1.73m^2^	82.3±15.5	84.5±16.7	87.9±17.5	81.5±17.4	0.08
Uric acid, mg/dL	4.2±0.9	4.4±0.9	4.3±1.1	4.8±1.3	<0.001
Glucose, mg/dL	92±15	91±11	88±11	91±11	0.42
Hemoglobin A1c, %	5.4±0.7	5.2±1.0	5.3±0.4	5.1±1.1	0.001
Framingham risk score, %	3.5±3.7	2.5±2.8	1.4±0.9	2.5±2.9	<0.001
Medical history, n (%)					
Hypertension	56 (14.4)	33 (13.8)	2 (4.0)	3 (13.6)	0.14
Dyslipidemia	118 (30.3)	55 (22.9)	6 (12.0)	5 (22.7)	0.012
Diabetes mellitus	11 (2.8)	5 (2.1)	1 (2.0)	2 (9.1)	0.45
Hyperuricemia	4 (1.0)	6 (2.5)	0 (0)	0 (0)	0.24
Current smoker, n (%)	1 (0.3)	5 (2.1)	1 (2.0)	1 (4.6)	0.07
Medication, n (%)					
RAS inhibitors	6 (1.5)	7 (2.9)	2 (4.0)	1 (4.5)	0.53
Beta-blockers	0 (0)	0 (0)	1 (2.0)	0 (0)	0.16
Calcium channel blockers	10 (2.6)	13 (5.4)	1 (2.0)	2 (9.1)	0.19
Statins	6 (1.5)	8 (3.3)	1 (2.0)	2 (9.1)	0.28
Antidiabetic drugs	4 (1.0)	2 (0.8)	0 (0)	0 (0)	0.45
Insulin	0 (0)	0 (0)	0 (0)	0 (0)	N/A

HDL indicates high-density lipoprotein; LDL, low-density lipoprotein; γ-GTP, gamma glutamyl transpeptidase; eGFR, estimated glomerular filtration rate; RAS, renin-angiotensin system; and N/A, not available.

We compared 227 light drinkers with 227 non-drinkers matched for age and medical histories. Clinical characteristics of the subjects in the two groups are summarized in Table 4. The concentrations of HDL cholesterol, γ-GTP, and uric acid were significantly higher in the light drinker group than in the non-drinker group. Heart rate was significantly lower in the light drinker group than in the non-drinker group. Other parameters were not significantly different between the two groups. There was no significant difference in FMD between non-drinkers and light drinkers (7.6±3.8% vs. 7.2±3.6%, P = 0.21).

**Table 4:** Clinical characteristics of the non-drinkers and light drinkers adjusted clinical status.

Variables	Alcohol consumption		P value
	None 0 g/week (n = 227)	Light 0 to <140 g/week (n = 227)	
Age, years	43±13	43±13	0.99
Body mass index, kg/m^2^	21.4±3.1	21.1±3.3	0.32
Systolic blood pressure, mm Hg	117±18	115±17	0.21
Diastolic blood pressure, mmHg	74±12	72±12	0.10
Heart rate, bpm	65±10	63±8	0.004
Total cholesterol, mg/dL	193±34	196±33	0.36
Triglycerides, mg/dL	74±40	73±45	0.78
HDL cholesterol, mg/dL	67±14	73±15	<0.001
LDL cholesterol, mg/dL	113±29	110±27	0.32
γ-GTP, mg/dL	18±15	23±26	0.02
eGFR, mL/min/1.73m^2^	83.2±14.8	83.9±16.5	0.65
Uric acid, mg/dL	4.1±0.8	4.4±0.9	<0.001
Glucose, mg/dL	89±12	91±11	0.13
Hemoglobin A1c, %	5.3±0.7	5.2±1.0	0.07
Framingham risk score, %	2.8±3.4	2.5±2.8	0.34
Medical history, n (%)			
Hypertension	25 (11.0)	29 (12.8)	0.56
Dyslipidemia	48 (21.2)	54 (23.8)	0.50
Diabetes mellitus	4 (1.8)	5 (2.2)	0.74
Hyperuricemia	0 (0)	6 (2.6)	N/A
Current smoker, n (%)	0 (0)	0 (0)	N/A
Medication, n (%)			
RAS inhibitors	3 (1.3)	7 (3.1)	0.41
Beta-blockers	0 (0)	1 (0.4)	N/A
Calcium channel blockers	1 (0.4)	10 (4.4)	0.01
Statins	4 (1.8)	8 (3.5)	0.47
Antidiabetic drugs	2 (0.9)	2 (0.9)	1.00
Insulin	0 (0)	0 (0)	N/A
Flow-mediated vasodilation, %	7.6±3.8	7.2±3.6	0.21

HDL indicates high-density lipoprotein; LDL, low-density lipoprotein; γ-GTP, gamma glutamyl transpeptidase; eGFR, estimated glomerular filtration rate; N/A, not available; and RAS, renin-angiotensin system.

We compared 50 moderate drinkers with 50 non-drinkers matched for age and medical histories. Clinical characteristics of the subjects in the two groups are summarized in Table 5. The concentrations of HDL cholesterol and γ-GTP were significantly higher in the moderate drinker group than in the non-drinker group. Other parameters were not significantly different between the two groups. There was no significant difference in FMD between non-drinkers and moderate drinkers (8.1±3.5% vs. 8.2±4.3%, P = 0.91).

**Table 5:** Clinical characteristics of the non-drinkers and moderate drinkers adjusted clinical status.

Variables	Alcohol consumption		P value
	None 0 g/week (n = 50)	Moderate 140< to 280 g/week (n = 50)	
Age, years	39±13	39±13	1.00
Body mass index, kg/m^2^	20.5±2.1	20.0±2.1	0.31
Systolic blood pressure, mm Hg	114±15	108±14	0.08
Diastolic blood pressure, mmHg	71±9	67±9	0.06
Heart rate, bpm	65±9	64±14	0.81
Total cholesterol, mg/dL	184±29	193±28	0.13
Triglycerides, mg/dL	67±28	67±38	0.90
HDL cholesterol, mg/dL	66±12	78±16	<0.001
LDL cholesterol, mg/dL	107±27	102±24	0.43
γ-GTP, mg/dL	15±6	28±33	0.009
eGFR, mL/min/1.73m^2^	86.3±13.0	87.9±17.5	0.63
Uric acid, mg/dL	4.2±0.9	4.3±1.1	0.75
Glucose, mg/dL	89±7	88±11	0.84
Hemoglobin A1c, %	5.2±0.8	5.3±0.4	0.60
Framingham risk score, %	2.0±2.3	1.4±0.9	0.11
Medical history, n (%)			
Hypertension	2 (4.0)	2 (4.0)	1.00
Dyslipidemia	6 (12.0)	6 (12.0)	1.00
Diabetes mellitus	1 (2.0)	1 (2.0)	1.00
Hyperuricemia	0 (0)	0 (0)	N/A
Current smoker, n (%)	1 (2.0)	1 (2.0)	1.00
Medication, n (%)			
RAS inhibitors	1 (2.0)	2 (4.0)	0.55
Beta-blockers	0 (0)	2 (4.0)	0.09
Calcium channel blockers	0 (0)	1 (2.0)	0.24
Statins	1 (2.0)	1 (2.0)	1.00
Antidiabetic drugs	0 (0)	0 (0)	N/A
Insulin	0 (0)	0 (0)	N/A
Flow-mediated vasodilation, %	8.1±3.5	8.2±4.3	0.91

HDL indicates high-density lipoprotein; LDL, low-density lipoprotein; γ-GTP, gamma glutamyl transpeptidase; eGFR, estimated glomerular filtration rate; N/A, not available; and RAS, renin-angiotensin system.

We compared 22 heavy drinkers with 22 non-drinkers matched for age and medical histories. Clinical characteristics of the subjects in the two groups are summarized in Table 6. The concentrations of HDL cholesterol and γ-GTP were significantly higher in the heavy drinker group than in the non-drinker group. Other parameters were not significantly different between the two groups. There was a significant difference in FMD between non-drinkers and heavy drinkers (8.9±3.5% vs. 5.9±2.5%, P = 0.002).

**Table 6:** Clinical characteristics of the non-drinkers and heavy drinkers adjusted clinical status.

Variables	Alcohol consumption		P value
	None 0 g/week (n = 22)	Heavy >280 g/week (n = 22)	
Age, years	42±17	42±17	1.00
Body mass index, kg/m^2^	20.9±1.7	20.8±2.6	0.90
Systolic blood pressure, mm Hg	119±17	117±24	0.81
Diastolic blood pressure, mmHg	73±9	71±12	0.41
Heart rate, bpm	68±11	68±11	0.87
Total cholesterol, mg/dL	183±30	193±27	0.25
Triglycerides, mg/dL	71±38	75±52	0.79
HDL cholesterol, mg/dL	66±13	78±17	0.016
LDL cholesterol, mg/dL	103±28	100±25	0.73
γ-GTP, mg/dL	16±7	30±22	0.009
eGFR, mL/min/1.73m^2^	84.2±18.0	81.5±17.4	0.62
Uric acid, mg/dL	4.2±0.9	4.8±1.3	0.10
Glucose, mg/dL	88±7	91±11	0.22
Hemoglobin A1c, %	5.5±0.6	5.1±1.1	0.25
Framingham risk score, %	2.8±2.9	2.5±2.9	0.80
Medical history, n (%)			
Hypertension	3 (13.6)	3 (13.6)	1.00
Dyslipidemia	6 (27.3)	6 (27.3)	1.00
Diabetes mellitus	2 (9.1)	2 (9.1)	1.00
Hyperuricemia	0 (0)	0 (0)	N/A
Current smoker, n (%)	1 (4.6)	1 (4.6)	1.00
Medication, n (%)			
RAS inhibitors	1 (4.6)	1 (4.6)	1.00
Beta-blockers	0 (0)	0 (0)	N/A
Calcium channel blockers	0 (0)	2 (9.1)	0.09
Statins	2 (9.1)	2 (9.1)	1.00
Antidiabetic drugs	0 (0)	0 (0)	N/A
Insulin	0 (0)	0 (0)	N/A
Flow-mediated vasodilation, %	8.9±3.5	5.9±2.5	0.002

HDL indicates high-density lipoprotein; LDL, low-density lipoprotein; γ-GTP, gamma glutamyl transpeptidase; eGFR, estimated glomerular filtration rate; N/A, not available; and RAS, renin-angiotensin system.

### Relationship between FMD and alcohol consumption in premenopausal women: A comparison of menstrual phase and follicular or luteal phase

The characteristics of the premenopausal women are summarized in S1 Table. There was no significant difference in FMD between women in the menstrual phase and women in the follicular or luteal phase (8.2±4.2% vs. 8.2±3.6%, P = 0.89). S1 Table shows the clinical characteristics of premenopausal women who were not in their menstrual period in accordance with alcohol consumption. There were significant differences in age, BMI, systolic blood pressure, diastolic pressure, HDL cholesterol, γ-GTP and uric acid among the four groups. Other parameters were not significantly different among the four groups. There was no significant difference in FMD between the non-drinker group and the drinker group (P = 0.07).

We compared 136 light drinkers with 136 non-drinkers matched for age and medical histories in premenopausal women who were not in their menstrual period. Clinical characteristics of the subjects in the two groups are shown in S3 Table. The concentrations of HDL cholesterol, γ-GTP, and uric acid were significantly higher in the light drinker group than in the non-drinker group. Diastolic blood pressure was significantly lower in the light drinker group than in the non-drinker group. Other parameters were not significantly different between the two groups. There was no significant difference in FMD between non-drinkers and light drinkers (8.3±3.5% vs. 7.9±3.4%, P = 0.38).

We compared 35 moderate drinkers with 35 non-drinkers matched for age and medical histories in the premenopausal women who were not in their menstrual period. Clinical characteristics of the subjects in the two groups are shown in S4 Table. The concentration of γ-GTP was significantly higher in the moderate drinker group than in the non-drinker group. Other parameters were not significantly different between the two groups. There was no significant difference in FMD between non-drinkers and moderate drinkers (8.9±3.2% vs. 9.1±4.3%, P = 0.86).

### Relationship between FMD and alcohol consumption in postmenopausal women

S5 Table shows the clinical characteristic of postmenopausal women in accordance with alcohol consumption. There were significant differences in systolic blood pressure and heart rate among the four groups. There was no significant difference in FMD among the four groups. There were five moderate drinkers and six heavy drinkers in the postmenopausal women. We compared 44 light drinkers with 44 non-drinkers matched for age and medical histories in postmenopausal women. Clinical characteristics of the subjects in the two groups are shown in S6 Table. Heart rate and concentration of triglycerides were significantly lower in the light drinker group than in the non-drinker group. Other parameters were not significantly different between the two groups. There was no significant difference in FMD between non-drinkers and light drinkers (6.1±2.9% vs. 5.3±3.1%, P = 0.21).

### Relationship between FMD and alcohol consumption in subjects who were not receiving drugs for hypertension, dyslipidemia and diabetes mellitus

S7 Table shows the clinical characteristic of subjects who were not receiving drugs for hypertension, dyslipidemia and diabetes mellitus. There were significant differences in age, BMI, systolic blood pressure, diastolic blood pressure, heart rate, triglycerides, HDL cholesterol, LDL cholesterol, eGFR, uric acid, hemoglobin A1c and FRS among the four groups. There was no significant difference in FMD among the four groups.

We compared 206 light drinkers with 206 non-drinkers matched for age and medical histories. Clinical characteristics of the subjects in the two groups are summarized in S8 Table. The concentrations of HDL cholesterol, γ-GTP, and uric acid were significantly higher in the light drinker group than in the non-drinker group. Heart rate and hemoglobin A1c level were significantly lower in the light drinker group than in the non-drinker group. Other parameters were not significantly different between the two groups. There was no significant difference in FMD between non-drinkers and light drinkers (7.9±3.9% vs. 7.3±3.5%, P = 0.14).

We compared 48 moderate drinkers with 48 non-drinkers matched for age and medical histories. Clinical characteristics of the subjects in the two groups are summarized in S9 Table. The concentrations of HDL cholesterol and γ-GTP were significantly higher in the moderate drinker group than in the non-drinker group. Other parameters were not significantly different between the two groups. There was no significant difference in FMD between non-drinkers and moderate drinkers (8.5±2.6% vs. 8.5±4.1%, P = 0.98).

We compared 20 heavy drinkers with 20 non-drinkers matched for age and medical histories. Clinical characteristics of the subjects in the two groups are summarized in S10 Table. The concentrations of HDL cholesterol, γ-GTP, and uric acid were significantly higher in the heavy drinker group than in the non-drinker group. Other parameters were not significantly different between the two groups. There was a significant difference in FMD between non-drinkers and heavy drinkers (8.8±3.3% vs. 6.5±1.8%, P = 0.007).

## Discussion

In the present study, we demonstrated that heavy alcohol consumption is associated with endothelial dysfunction but that light to moderate alcohol consumption is not associated with endothelial dysfunction in women. Our study is the first study to show a relationship between alcohol consumption and endothelial function assessed by FMD in a general population consisting of only women.

Previous studies showed that heavy alcohol drinking is associated with risk of all-cause death and cardiovascular events in women [4–6]. On the other hand, light to moderate alcohol drinking reduces the incidence of all-cause death and cardiovascular events in women [4–6]. Several lines of evidence have shown that FMD is a predictor of cardiovascular events. In addition, several meta-analyses have revealed the results of multivariant analysis of hazard ratios in studies showing an association between coronary or peripheral endothelial function and cardiovascular events. It is expected that an increase in or augmentation of FMD contributes to the decrease in cardiovascular events in women who are light to moderate alcohol drinkers. However, in the present study, endothelial function was not improved in light to moderate drinkers in women. It is well known that light to moderate alcohol intake has beneficial effects, including anti-thrombosis and increase in estrogen, on prevention of cardiovascular events. Antithrombotic effects of alcohol other than improvement of endothelial function may be associated with the reduction in cardiovascular events in light to moderate drinkers in women.

In the present study, light to moderate alcohol intake was not associated with endothelial dysfunction in women, while a previous study showed that light to moderate alcohol intake is associated with endothelial dysfunction in men [24]. Suzuki et al. reported that moderate alcohol intake improved endothelial function assessed by FMD in 384 men and 500 women [30]. However, the participants in that study were older and at higher risk of endothelial dysfunction than the subjects in our study; 603 (68.2%) of the participants in that study had hypertension, 202 (22.9%) had diabetes and 435 (43.5%) had dyslipidemia. The authors did not state the number of heavy drinking women. It is not clear whether women are more vulnerable than men to the effects of alcohol when drinking a light to moderate amount of alcohol.

Unfortunately, in the present study, multiple regression analysis of FMD in heavy drinkers and non-drinkers could not be performed due to the small number of subjects. Thus, we compared 22 heavy drinkers with 22 non-drinkers matched for age and medical histories. FMD was significantly lower in the heavy drinkers than in the non-drinkers. In the same way, we showed that there was no significant difference in FMD between 50 moderate drinkers and 50 selected non-drinkers. Suzuki et al. also showed that heavy alcohol intake was significantly associated with endothelial dysfunction in their subjects [30]. Di Gennaro C et al. reported that FMD was significantly lower in 29 heavy alcoholics than in teetotalers (8.5±5.4% vs. 14.9±7.4%, P<0.001), but there were only two women alcoholics in their study [31]. These findings suggest that heavy alcohol drinking has a harmful effect on endothelial function in women.

A balance of nitric oxide (NO) and oxidative stress plays an important role in the maintenance of healthy endothelial function. Several investigators have shown that alcohol intake increases vasodilators, vasoconstrictors, oxidative stress, and thrombotic factors in a dose-dependent manner [32, 33]. Soardo et al. reported that cultured human aorta endothelial cells exposed to alcohol at a dose being equal to heavy drinking in humans released both the vasoconstrictor endothelin-1 and the vasodilator NO and increased the concentration of the oxidative stress marker malondialdehyde and decreased the concentration of the antioxidant marker intracellular glutathione [32]. In a previous study, we confirmed that heavy drinking is associated with endothelial dysfunction in men [24]. Under the condition of heavy drinking, it is likely that vasoconstrictors and oxidative stress predominately act in the vasculature, resulting in endothelial dysfunction in both men and women.

On the other hand, under the condition of light to moderate drinking, it is thought that a balance of NO and oxidative stress is maintained. Interestingly, it has been shown that microvascular function of isolated subcutaneous adipose arterioles is maintained in moderate drinkers compared with those in alcohol abstainers and is improved by treatment with tetrahydrobiopterin in moderate drinkers but not in alcohol abstainers [34]. However, the mechanisms related to changes in FMD for heavy drinking but not moderate drinking are unclear. Alcohol intake may be a double-edged sword for endothelial function.

Our study has several limitations. First, there is potential for bias because the grams of alcohol consumption were calculated using self-reported alcohol habits. In the present study, alcohol consumption significantly correlated with HDL cholesterol, γ-GTP and uric acid concentrations, suggesting that alcohol consumption estimated by self-reported alcohol habits reflects an accurate amount of alcohol intake. Second, we evaluated the association between the amount of ethanol and FMD. However, we did not evaluate the influence of the kind of alcohol beverage and drinking pattern on FMD. Third, it was shown that the menstrual cycle and polycystic ovarian syndrome affected endothelial function [35–37]. FMD has been reported to vary during the menstrual cycle, with significant increases from the follicular to luteal phases, sharp falls in the early luteal phase, and significant recoveries in the luteal phase [35, 36]. In the present study, FMD values were similar in the follicular and luteal phases and the menstrual phase. By way of precaution, we evaluated the relationship between FMD and alcohol consumption in 372 premenopausal women who were not in their menstrual period, and we confirmed that light to moderate alcohol consumption did not alter endothelial function. We extracted postmenopausal women to evaluate the association of FMD with alcohol consumption. There was also no significant difference in FMD between non-drinkers and light drinkers in postmenopausal women. However, we had no information on the history of polycystic ovarian syndrome. In addition, the number of subjects was small for evaluating the relationship between alcohol consumption and endothelial function according to the menstrual cycle phase. Further studies are needed to confirm the effects of the menstrual cycle and polycystic ovarian syndrome on the relationship between FMD and alcohol consumption in a larger population. Fourth, since carrying out case-control comparisons in small numbers of non-drinkers matched to either moderate drinkers or heavy drinkers for the prevalence of these disorders does not rule out any confounding by background treatment, we analyzed the relationship between alcohol consumption and endothelial function in women who were not receiving treatment for dyslipidemia, hypertension or diabetes mellitus. We confirmed that heavy alcohol consumption is associated with endothelial dysfunction but that moderate alcohol consumption is not associated with endothelial dysfunction in subjects who were not receiving treatment for dyslipidemia, hypertension or diabetes mellitus. However, we cannot deny the possibility of a Type II error in interpreting the results. Fifth, in a previous study, we showed that FMD was lowered even in light drinking and moderate drinking men compared with that in non-drinkers [24]. It has been shown that alcohol intake increases estrogen concentration in both premenopausal women and postmenopausal women [38, 39]. In addition, Vatsalya et al. showed that alcohol intake increases estradiol levels in women and decreases estradiol levels in men [40]. One possible reason for differences in FMD between men and women is the effects of estrogen. However, the precise reasons for the difference in effects of light to moderate alcohol intake on endothelial function between men and women remain unclear. Sixth, we had no information on exercise and diet of the subjects. Exercise and diet are factors affecting both alcohol consumption and endothelial function [41, 42]. We cannot rule out the possibility that exercise and diet influence the association between alcohol consumption and endothelial function. Finally, measurements of nitroglycerine-induced vasodilation as an index of endothelium-independent vasodilation would enable more specific conclusions concerning the role of alcohol consumption in vascular function to be drawn.

## Conclusions

Light to moderate alcohol intake is not associated with endothelial dysfunction in women. On the other hand, heavy alcohol intake is associated with endothelial dysfunction in women.

## Supporting information

S1 Fig Participant flowchart. (TIF)

S1 Table Clinical characteristics of premenopausal women. (DOCX)

S2 Table Clinical characteristics of premenopausal women who were not in their menstrual period. (DOCX)

S3 Table Clinical characteristics of non-drinkers and light drinkers with adjusted clinical status in premenopausal women who were not in their menstrual period. (DOCX)

S4 Table Clinical characteristics of non-drinkers and moderate drinkers with adjusted clinical status in premenopausal women who were not in their menstrual period. (DOCX)

S5 Table Clinical characteristics of postmenopausal women in accordance with alcohol consumption. (DOCX)

S6 Table Clinical characteristics of non-drinkers and light drinkers with adjusted clinical status in postmenopausal women. (DOCX)

S7 Table Clinical characteristics of subjects who were not receiving drugs for hypertension, dyslipidemia and diabetes mellitus in accordance with alcohol consumption. (DOCX)

S8 Table Clinical characteristics of non-drinkers and light drinkers with adjusted clinical status in subjects who were not receiving drugs for hypertension, dyslipidemia and diabetes mellitus. (DOCX)

S9 Table Clinical characteristics of non-drinkers and moderate drinkers with adjusted clinical status in subjects who were not receiving drugs for hypertension, dyslipidemia and diabetes mellitus. (DOCX)

S10 Table Clinical characteristics of non-drinkers and heavy drinkers with adjusted clinical status in subjects who were not receiving drugs for hypertension, dyslipidemia and diabetes mellitus. (DOCX)

S1 File Alcohol consumption questionnaire. (DOCX)
