# Supplementary material for: Endothelial function is preserved in light to moderate alcohol drinkers but is impaired in heavy drinkers in women: Flow-mediated Dilation Japan (FMD-J) study
Source: PLoS One. 2020 Dec 3;15(12):e0243216. doi: 10.1371/journal.pone.0243216 (PMC7714190; doi:10.1371/journal.pone.0243216)
Supplement: S1 Table — (DOCX) [file pone.0243216.s002.docx]

**S1 Table**. Clinical characteristics of the premenopausal women

| Variables | Menstrual phase  (n=68) | Follicular or luteal phase  (n=372) | P value |
| --- | --- | --- | --- |
| Age, yr | 35±10 | 38±9 | 0.08 |
| Body mass index, kg/m^2^ | 21.3±4.5 | 20.8±3.0 | 0.22 |
| Systolic blood pressure, mm Hg | 112±15 | 112±14 | 0.79 |
| Diastolic blood pressure, mmHg | 72±10 | 70±11 | 0.20 |
| Heart rate, bpm | 65±8 | 64±10 | 0.48 |
| Total cholesterol, mg/dL | 178±31 | 188±31 | 0.02 |
| Triglycerides, mg/dL | 72±45 | 66±37 | 0.26 |
| HDL cholesterol, mg/dL | 65±16 | 71±14 | 0.006 |
| LDL cholesterol, mg/dL | 101±28 | 107±27 | 0.14 |
| γ-GTP, mg/dL | 23±33 | 19±18 | 0.20 |
| eGFR, mL/min/1.73m^2^ | 88.8±14.4 | 88.1±15.9 | 0.72 |
| Uric acid, mg/dL | 4.1±0.9 | 4.2±0.9 | 0.64 |
| Glucose, mg/dL | 88±8 | 89±10 | 0.33 |
| Hemoglobin A1c, % | 5.1±0.9 | 5.2±0.9 | 0.82 |
| Framingham risk score, % | 1.6±1.7 | 1.6±1.5 | 0.66 |
| Medical history, n (%) |  |  |  |
| Hypertension | 2 (2.9) | 16 (4.3) | 0.59 |
| Dyslipidemia | 10 (14.7) | 49 (13.2) | 0.74 |
| Diabetes mellitus | 0 (0) | 2 (0.5) | 0.41 |
| Hyperuricemia | 0 (0) | 1 (0.3) | 0.56 |
| Current smoker, n (%) | 0 (0) | 3 (0.7) | 0.31 |
| Medication, n (%) |  |  |  |
| RAS inhibitors | 0 (0) | 2 (0.5) | 0.41 |
| Beta-blockers | 0 (0) | 0 (0) | N/A |
| Calcium channel blockers | 1 (1.5) | 2 (0.5) | 0.45 |
| Statins | 0 (0) | 2 (0.5) | 0.41 |
| Antidiabetic drugs | 0 (0) | 2 (0.5) | 0.41 |
| Insulin | 0 (0) | 0 (0) | N/A |
| Flow-mediated vasodilation, % | 8.2±4.2 | 8.2±3.6 | 0.89 |

HDL indicates high-density lipoprotein; LDL, low-density lipoprotein; γ-GTP, gamma glutamyl transpeptidase; eGFR, estimated glomerular filtration rate; RAS, renin angiotensin system; and N/A, not available.
