# Supplementary material for: Endothelial function is preserved in light to moderate alcohol drinkers but is impaired in heavy drinkers in women: Flow-mediated Dilation Japan (FMD-J) study
Source: PLoS One. 2020 Dec 3;15(12):e0243216. doi: 10.1371/journal.pone.0243216 (PMC7714190; doi:10.1371/journal.pone.0243216)
Supplement: S2 Table — (DOCX) [file pone.0243216.s003.docx]

**S2 Table**. Clinical characteristics of premenopausal women who were not in their menstrual period

| Variables | Total  (n=372) | Alcohol consumption | | | | P value for trend |
| --- | --- | --- | --- | --- | --- | --- |
|  |  | None  0  g/week  (n=176) | Light  0< to 140 g/week  (n=149) | Moderate  140< to 280 g/week  (n=35) | Heavy  >280 g/week  (n=12) |  |
| Age, yr | 38±9 | 39±7 | 37±7 | 35±9 | 33±11 | 0.03 |
| Body mass index, kg/m^2^ | 20.8±3.0 | 21.2±3.3 | 20.4±2.8 | 20.0±2.3 | 20.2±1.5 | 0.03 |
| Systolic blood pressure, mm Hg | 112±14 | 114±14 | 111±15 | 106±12 | 108±9 | 0.02 |
| Diastolic blood pressure, mm Hg | 70±11 | 72±11 | 69±12 | 66±8 | 65±5 | 0.003 |
| Herat rate, bpm | 64±10 | 65±9 | 63±8 | 64±16 | 66±7 | 0.33 |
| Total cholesterol, mg/dL | 188±31 | 187±31 | 189±31 | 190±30 | 184±21 | 0.90 |
| Triglycerides, mg/dL | 66±37 | 67±30 | 68±44 | 60±38 | 55±19 | 0.52 |
| HDL cholesterol, mg/dL | 71±14 | 67±13 | 72±15 | 78±16 | 76±13 | <0.001 |
| LDL cholesterol, mg/dL | 107±27 | 110±28 | 105±25 | 101±26 | 99±21 | 0.16 |
| γ-GTP, mg/dL | 19±18 | 17±9 | 20±24 | 24±20 | 29±26 | 0.02 |
| eGFR, mL/min/1.73m^2^ | 88.1±15.9 | 87.0±14.7 | 88.9±17.2 | 89.6±16.9 | 88.7±14.7 | 0.70 |
| Uric acid, mg/dL | 4.2±0.9 | 4.0±0.8 | 4.3±0.9 | 4.3±1.0 | 4.7±1.7 | 0.001 |
| Glucose, mg/dL | 89±10 | 89±9 | 89±9 | 87±12 | 91±8 | 0.63 |
| Hemoglobin A1c, % | 5.2±0.9 | 5.3±0.7 | 5.0±1.2 | 5.3±0.3 | 5.3±0.2 | 0.06 |
| Framingham risk scores, % | 1.6±1.5 | 1.6±1.4 | 1.6±1.8 | 1.2±0.8 | 1.3±0.9 | 0.50 |
| Medical history, n (%) |  |  |  |  |  |  |
| Hypertension | 16 (4.3) | 5 (2.8) | 11 (7.4) | 0 (0) | 0 (0) | 0.046 |
| Dyslipidemia | 49 (13.2) | 24 (13.6) | 22 (14.8) | 3 (8.6) | 0 (0) | 0.22 |
| Diabetes mellitus | 2 (0.5) | 1 (0.6) | 1 (0.7) | 0 (0) | 0 (0) | 0.91 |
| Hyperuricemia | 1 (0.3) | 0 (0) | 1 (0.7) | 0 (0) | 0 (0) | 0.65 |
| Current smoker, n (%) | 3 (0.8) | 0 (0) | 2 (1.3) | 1 (2.9) | 0 (0) | 0.20 |
| Medication, n (%) |  |  |  |  |  |  |
| RAS inhibitors | 2 (0.5) | 0 (0) | 2 (1.4) | 0 (0) | 0 (0) | 0.30 |
| Beta-blockers | 0 (0) | 0 (0) | 0 (0) | 0 (0) | 0 (0) | N/A |
| Calcium channel blockers | 2 (0.5) | 0 (0) | 2 (1.4) | 0 (0) | 0 (0) | 0.30 |
| Statins | 2 (0.5) | 1 (0.6) | 1 (0.7) | 0 (0) | 0 (0) | 0.90 |
| Antidiabetic drugs | 2 (0.8) | 1 (1.3) | 1 (0.9) | 0 (0) | 0 (0) | 0.82 |
| Insulin | 0 (0) | 0 (0) | 0 (0) | 0 (0) | 0 (0) | N/A |
| Flow-mediated vasodilation, % | 8.2±3.6 | 8.3±3.6 | 8.0±3.5 | 9.1±4.3 | 6.1±2.0 | 0.07 |

HDL indicates high-density lipoprotein; LDL, low-density lipoprotein; γ-GTP, gamma glutamyl transpeptidase; eGFR, estimated glomerular filtration rate; RAS, renin angiotensin system; and N/A, not available.
