# Supplementary material for: Endothelial function is preserved in light to moderate alcohol drinkers but is impaired in heavy drinkers in women: Flow-mediated Dilation Japan (FMD-J) study
Source: PLoS One. 2020 Dec 3;15(12):e0243216. doi: 10.1371/journal.pone.0243216 (PMC7714190; doi:10.1371/journal.pone.0243216)
Supplement: S3 Table — (DOCX) [file pone.0243216.s004.docx]

**S3 Table.** Clinical characteristics of non-drinkers and light drinkers with adjusted clinical status in premenopausal women who were not in their menstrual period

| Variables | Alcohol consumption | | P value |
| --- | --- | --- | --- |
|  | None  0 g/week  (n=136) | Light  0 to <140 g/week  (n=136) |  |
| Age, years | 38±9 | 37±9 | 0.64 |
| Body mass index, kg/m^2^ | 20.5±2.7 | 20.4±2.8 | 0.76 |
| Systolic blood pressure, mm Hg | 112±14 | 109±13 | 0.13 |
| Diastolic blood pressure, mmHg | 70±11 | 68±11 | 0.03 |
| Heart rate, bpm | 64±9 | 63±8 | 0.08 |
| Total cholesterol, mg/dL | 186±31 | 187±31 | 0.95 |
| Triglycerides, mg/dL | 64±30 | 64±32 | 0.88 |
| HDL cholesterol, mg/dL | 68±12 | 72±13 | 0.01 |
| LDL cholesterol, mg/dL | 108±27 | 103±25 | 0.14 |
| γ-GTP, mg/dL | 16±8 | 18±9 | 0.04 |
| eGFR, mL/min/1.73m^2^ | 87.4±15.9 | 88.5±16.6 | 0.59 |
| Uric acid, mg/dL | 3.9±0.8 | 4.3±0.9 | <0.001 |
| Glucose, mg/dL | 88±9 | 89±9 | 0.66 |
| Hemoglobin A1c, % | 5.2±0.8 | 5.0±1.1 | 0.09 |
| Framingham risk score, % | 1.5±1.4 | 1.4±1.4 | 0.46 |
| Medical history, n (%) |  |  |  |
| Hypertension | 5 (3.7) | 4 (2.9) | 0.73 |
| Dyslipidemia | 17 (12.5) | 16 (11.8) | 0.85 |
| Diabetes mellitus | 1 (0.7) | 1 (0.7) | 1.00 |
| Hyperuricemia | 0 (0) | 1 (0.7) | N/A |
| Current smoker, n (%) | 0 (0) | 0 (0) | N/A |
| Medication, n (%) |  |  |  |
| RAS inhibitors | 0 (0) | 2 (1.5) | N/A |
| Beta-blockers | 0 (0) | 0 (0) | N/A |
| Calcium channel blockers | 0 (0) | 2 (1.5) | N/A |
| Statins | 0 (0) | 0 (0) | N/A |
| Antidiabetic drugs | 1 (0.7) | 1 (0.7) | 1.00 |
| Insulin | 0 (0) | 0 (0) | N/A |
| Flow-mediated vasodilation, % | 8.3±3.5 | 7.9±3.4 | 0.38 |

HDL indicates high-density lipoprotein; LDL, low-density lipoprotein; γ-GTP, gamma glutamyl transpeptidase; eGFR, estimated glomerular filtration rate; N/A, not available; and RAS, renin-angiotensin system.
