# Supplementary material for: Endothelial function is preserved in light to moderate alcohol drinkers but is impaired in heavy drinkers in women: Flow-mediated Dilation Japan (FMD-J) study
Source: PLoS One. 2020 Dec 3;15(12):e0243216. doi: 10.1371/journal.pone.0243216 (PMC7714190; doi:10.1371/journal.pone.0243216)
Supplement: S4 Table — (DOCX) [file pone.0243216.s005.docx]

**S4 Table**. Clinical characteristics of non-drinkers and moderate drinkers with adjusted clinical status in premenopausal women who were not in their menstrual period

| Variables | Alcohol consumption | | P value |
| --- | --- | --- | --- |
|  | None  0 g/week  (n=35) | Moderate  140< to 280 g/week  (n=35) |  |
| Age, yr | 35±8 | 35±8 | 0.99 |
| Body mass index, kg/m^2^ | 20.6±2.6 | 20.0±2.3 | 0.31 |
| Systolic blood pressure, mm Hg | 109±11 | 106±12 | 0.77 |
| Diastolic blood pressure, mmHg | 68±9 | 66±8 | 0.35 |
| Heart rate, bpm | 65±10 | 64±16 | 0.92 |
| Total cholesterol, mg/dL | 182±31 | 190±30 | 0.28 |
| Triglycerides, mg/dL | 51±19 | 60±38 | 0.19 |
| HDL cholesterol, mg/dL | 72±12 | 78±16 | 0.10 |
| LDL cholesterol, mg/dL | 103±28 | 101±26 | 0.80 |
| γ-GTP, mg/dL | 16±6 | 24±20 | 0.03 |
| eGFR, mL/min/1.73m^2^ | 88.8±14.5 | 90.0±16.9 | 0.84 |
| Uric acid, mg/dL | 4.1±0.9 | 4.3±1.0 | 0.46 |
| Glucose, mg/dL | 87±10 | 87±12 | 0.88 |
| Hemoglobin A1c, % | 5.3±0.3 | 5.3±0.3 | 0.49 |
| Framingham risk score, % | 1.3±1.1 | 1.2±0.8 | 0.90 |
| Medical history, n (%) |  |  |  |
| Hypertension | 0 (0) | 0 (0) | N/A |
| Dyslipidemia | 3 (8.6) | 3 (8.6) | 1.00 |
| Diabetes mellitus | 0 (0) | 0 (0) | N/A |
| Hyperuricemia | 0 (0) | 0 (0) | N/A |
| Current smoker, n (%) | 0 (0) | 1 (2.9) | 0.24 |
| Medication, n (%) |  |  |  |
| RAS inhibitors | 0 (0) | 0 (0) | N/A |
| Beta-blockers | 0 (0) | 0 (0) | N/A |
| Calcium channel blockers | 0 (0) | 0 (0) | N/A |
| Statins | 0 (0) | 0 (0) | N/A |
| Antidiabetic drugs | 0 (0) | 0 (0) | N/A |
| Insulin | 0 (0) | 0 (0) | N/A |
| Flow-mediated vasodilation, % | 8.9±3.2 | 9.1±4.3 | 0.86 |

HDL indicates high-density lipoprotein; LDL, low-density lipoprotein; γ-GTP, gamma glutamyl transpeptidase; eGFR, estimated glomerular filtration rate; N/A, not available; and RAS, renin-angiotensin system.
