# Supplementary material for: Endothelial function is preserved in light to moderate alcohol drinkers but is impaired in heavy drinkers in women: Flow-mediated Dilation Japan (FMD-J) study
Source: PLoS One. 2020 Dec 3;15(12):e0243216. doi: 10.1371/journal.pone.0243216 (PMC7714190; doi:10.1371/journal.pone.0243216)
Supplement: S5 Table — (DOCX) [file pone.0243216.s006.docx]

**S5 Table**. Clinical characteristics of the postmenopausal women in accordance with alcohol consumption

| Variables | Total  (n=172) | Alcohol consumption | | | | P value for trend |
| --- | --- | --- | --- | --- | --- | --- |
|  |  | None  0  g/week  (n=115) | Light  0< to 140 g/week  (n=46) | Moderate  140< to 280 g/week  (n=5) | Heavy  >280 g/week  (n=6) |  |
| Age, yr | 62±7 | 63±7 | 61±7 | 69±9 | 63±11 | 0.13 |
| Body mass index, kg/m^2^ | 22.6±3.4 | 22.6±3.3 | 22.8±3.9 | 21.0±2.1 | 22.5±4.2 | 0.76 |
| Systolic blood pressure, mm Hg | 132±19 | 134±19 | 126±16 | 119±24 | 144±31 | 0.02 |
| Diastolic blood pressure, mm Hg | 80±11 | 81±11 | 78±11 | 73±14 | 84±12 | 0.32 |
| Herat rate, bpm | 67±11 | 68±12 | 64±8 | 67±10 | 77±14 | 0.02 |
| Total cholesterol, mg/dL | 222±32 | 224±32 | 219±33 | 204±23 | 211±38 | 0.39 |
| Triglycerides, mg/dL | 106±63 | 111±67 | 93±51 | 86±31 | 111±73 | 0.39 |
| HDL cholesterol, mg/dL | 70±17 | 68±15 | 75±19 | 70±21 | 71±20 | 0.17 |
| LDL cholesterol, mg/dL | 132±30 | 136±31 | 128±26 | 117±25 | 115±29 | 0.11 |
| γ-GTP, mg/dL | 26±21 | 25±17 | 30±29 | 29±17 | 27±14 | 0.53 |
| eGFR, mL/min/1.73m^2^ | 73.4±13.6 | 74.0±13.8 | 73.3±12.3 | 69.9±16.2 | 65.0±16.5 | 0.42 |
| Uric acid, mg/dL | 4.6±1.0 | 4.5±1.0 | 4.8±1.0 | 3.9±1.4 | 5.0±0.5 | 0.11 |
| Glucose, mg/dL | 97±19 | 97±21 | 97±13 | 96±5 | 99±14 | 0.99 |
| Hemoglobin A1c, % | 5.7±0.5 | 5.7±0.5 | 5.6±0.5 | 5.7±0.7 | 5.7±0.6 | 0.55 |
| Framingham risk scores, % | 6.7±4.2 | 7.2±4.4 | 5.8±3.6 | 3.0±1.4 | 6.8±3.0 | 0.09 |
| Medical history, n (%) |  |  |  |  |  |  |
| Hypertension | 61 (35.7) | 42 (36.8) | 14 (30.4) | 2 (40.0) | 3 (50.0) | 0.76 |
| Dyslipidemia | 97 (56.7) | 66 (57.9) | 25 (54.4) | 2 (40.0) | 4 (66.7) | 0.81 |
| Diabetes mellitus | 15 (8.8) | 9 (7.9) | 3 (6.5) | 1 (20.0) | 2 (33.3) | 0.28 |
| Hyperuricemia | 7 (4.1) | 4 (3.5) | 3 (6.5) | 0 (0) | 0 (0) | 0.65 |
| Current smoker, n (%) | 2 (1.2) | 0 (0) | 1 (2.2) | 0 (0) | 1 (16.7) | 0.08 |
| Medication, n (%) |  |  |  |  |  |  |
| RAS inhibitors | 12 (7.7) | 5 (5.0) | 4 (9.3) | 2 (40.0) | 1 (16.7) | 0.12 |
| Beta-blockers | 1 (0.1) | 0 (0) | 0 (0) | 1 (20.0) | 0 (0) | 0.07 |
| Calcium channel blockers | 17 (11.0) | 8 (7.9) | 6 (14.9) | 1 (20.0) | 2 (33.3) | 0.27 |
| Statins | 13 (10.9) | 4 (5.2) | 6 (18.2) | 1 (25.0) | 2 (40.0) | 0.04 |
| Antidiabetic drugs | 2 (2.4) | 2 (3.5) | 0 (0) | 0 (0) | 0 (0) | 0.67 |
| Insulin | 0 (0) | 0 (0) | 0 (0) | 0 (0) | 0 (0) | N/A |
| Flow-mediated vasodilation, % | 5.3±3.2 | 5.4±3.3 | 5.2±3.1 | 3.7±3.3 | 4.3±3.3 | 0.55 |

HDL indicates high-density lipoprotein; LDL, low-density lipoprotein; γ-GTP, gamma glutamyl transpeptidase; eGFR, estimated glomerular filtration rate; RAS, renin angiotensin system; and N/A, not available.
