# Supplementary material for: Endothelial function is preserved in light to moderate alcohol drinkers but is impaired in heavy drinkers in women: Flow-mediated Dilation Japan (FMD-J) study
Source: PLoS One. 2020 Dec 3;15(12):e0243216. doi: 10.1371/journal.pone.0243216 (PMC7714190; doi:10.1371/journal.pone.0243216)
Supplement: S6 Table — (DOCX) [file pone.0243216.s007.docx]

**S6 Table.** Clinical characteristics of non-drinkers and light drinkers with adjusted clinical status in postmenopausal women

| Variables | Alcohol consumption | | P value |
| --- | --- | --- | --- |
|  | None  0 g/week  (n=44) | Light  0 to <140 g/week  (n=44) |  |
| Age, years | 61±7 | 61±7 | 0.89 |
| Body mass index, kg/m^2^ | 22.9±3.0 | 22.7±3.9 | 0.87 |
| Systolic blood pressure, mm Hg | 132±17 | 127±17 | 0.14 |
| Diastolic blood pressure, mmHg | 81±11 | 79±12 | 0.25 |
| Heart rate, bpm | 68±12 | 63±8 | 0.03 |
| Total cholesterol, mg/dL | 227±31 | 219±34 | 0.27 |
| Triglycerides, mg/dL | 125±73 | 94±52 | 0.03 |
| HDL cholesterol, mg/dL | 68±16 | 74±19 | 0.08 |
| LDL cholesterol, mg/dL | 137±29 | 128±26 | 0.10 |
| γ-GTP, mg/dL | 24±14 | 30±30 | 0.18 |
| eGFR, mL/min/1.73m^2^ | 76.4±13.8 | 73.6±12.3 | 0.33 |
| Uric acid, mg/dL | 5.8±0.6 | 5.6±0.5 | 0.13 |
| Glucose, mg/dL | 100±26 | 96±13 | 0.45 |
| Hemoglobin A1c, % | 5.2±0.8 | 5.0±1.1 | 0.09 |
| Framingham risk score, % | 7.0±3.7 | 5.9±3.6 | 0.20 |
| Medical history, n (%) |  |  |  |
| Hypertension | 12 (27.3) | 13 (29.6) | 0.81 |
| Dyslipidemia | 31 (70.5) | 24 (54.6) | 0.12 |
| Diabetes mellitus | 4 (9.0) | 3 (6.8) | 0.69 |
| Hyperuricemia | 2 (4.6) | 3 (6.8) | 0.64 |
| Current smoker, n (%) | 0 (0) | 0 (0) | N/A |
| Medication, n (%) |  |  |  |
| RAS inhibitors | 2 (4.5) | 4 (9.1) | 0.39 |
| Beta-blockers | 0 (0) | 0 (0) | N/A |
| Calcium channel blockers | 2 (4.5) | 5 (11.4) | 0.23 |
| Statins | 1 (2.3) | 6 (13.6) | 0.06 |
| Antidiabetic drugs | 1 (2.3) | 0 (0) | N/A |
| Insulin | 0 (0) | 0 (0) | N/A |
| Flow-mediated vasodilation, % | 6.1±2.9 | 5.3±3.1 | 0.21 |

HDL indicates high-density lipoprotein; LDL, low-density lipoprotein; γ-GTP, gamma glutamyl transpeptidase; eGFR, estimated glomerular filtration rate; N/A, not available; and RAS, renin-angiotensin system.
