# Supplementary material for: Endothelial function is preserved in light to moderate alcohol drinkers but is impaired in heavy drinkers in women: Flow-mediated Dilation Japan (FMD-J) study
Source: PLoS One. 2020 Dec 3;15(12):e0243216. doi: 10.1371/journal.pone.0243216 (PMC7714190; doi:10.1371/journal.pone.0243216)
Supplement: S7 Table — (DOCX) [file pone.0243216.s008.docx]

**S7 Table**. Clinical characteristics of the subjects who were not receiving drugs for hypertension, dyslipidemia and diabetes mellitus in accordance with alcohol consumption

| Variables | Total  (n=644) | Alcohol consumption | | | | P value for trend |
| --- | --- | --- | --- | --- | --- | --- |
|  |  | None  0  g/week  (n=358) | Light  0< to 140 g/week  (n=218) | Moderate  140< to 280 g/week  (n=48) | Heavy  >280 g/week  (n=20) |  |
| Age, yr | 43±12 | 45±13 | 42±12 | 38±11 | 38±13 | <0.001 |
| Body mass index, kg/m^2^ | 21.2±3.2 | 21.7±3.6 | 20.7±2.9 | 20.0±2.0 | 20.3±1.8 | <0.001 |
| Systolic blood pressure, mm Hg | 116±16 | 119±17 | 113±16 | 107±13 | 113±17 | <0.001 |
| Diastolic blood pressure, mm Hg | 73±11 | 75±11 | 71±12 | 67±9 | 70±11 | <0.001 |
| Herat rate, bpm | 64±10 | 66±10 | 62±8 | 64±14 | 67±11 | 0.001 |
| Total cholesterol, mg/dL | 197±35 | 199±37 | 195±33 | 193±28 | 195±27 | 0.43 |
| Triglycerides, mg/dL | 76±46 | 80±47 | 71±44 | 66±38 | 72±54 | 0.03 |
| HDL cholesterol, mg/dL | 70±15 | 67±14 | 73±15 | 79±16 | 79±16 | <0.001 |
| LDL cholesterol, mg/dL | 114±30 | 118±32 | 109±27 | 102±25 | 100±26 | <0.001 |
| γ-GTP, mg/dL | 22±23 | 20±18 | 23±26 | 27±33 | 31±23 | 0.05 |
| eGFR, mL/min/1.73m^2^ | 84.4±15.7 | 82.8±15.4 | 85.9±16.2 | 88.9±16.6 | 84.5±15.4 | 0.02 |
| Uric acid, mg/dL | 4.3±0.9 | 4.1±0.9 | 4.4±0.9 | 4.2±1.0 | 4.8±1.4 | 0.001 |
| Glucose, mg/dL | 90±12 | 91±14 | 90±9 | 88±11 | 91±12 | 0.57 |
| Hemoglobin A1c, % | 5.3±0.8 | 5.4±0.7 | 5.1±0.9 | 5.3±0.3 | 5.0±1.1 | 0.001 |
| Framingham risk scores, % | 2.6±2.9 | 3.1±3.3 | 2.2±2.5 | 1.4±0.9 | 2.5±2.9 | <0.001 |
| Medical history, n (%) |  |  |  |  |  |  |
| Hypertension | 43 (6.7) | 28 (7.8) | 14 (6.4) | 0 (0) | 1 (0.1) | 0.06 |
| Dyslipidemia | 146 (22.7) | 96 (26.8) | 42 (19.3) | 5 (10.4) | 3 (15.0) | 0.014 |
| Diabetes mellitus | 3 (0.5) | 2 (0.6) | 1 (0.5) | 0 (0) | 0 (0) | 0.87 |
| Hyperuricemia | 5 (0.8) | 3 (0.8) | 2 (0.9) | 0 (0) | 0 (0) | 0.77 |
| Current smoker, n (%) | 6 (0.9) | 1 (0.3) | 3 (1.4) | 1 (2.1) | 1 (5.0) | 0.17 |
| Flow-mediated vasodilation, % | 7.5±3.6 | 7.5±3.7 | 7.4±3.5 | 8.5±4.1 | 6.5±1.8 | 0.16 |

HDL indicates high-density lipoprotein; LDL, low-density lipoprotein; γ-GTP, gamma glutamyl transpeptidase; and eGFR, estimated glomerular filtration rate.
