# Supplementary material for: Endothelial function is preserved in light to moderate alcohol drinkers but is impaired in heavy drinkers in women: Flow-mediated Dilation Japan (FMD-J) study
Source: PLoS One. 2020 Dec 3;15(12):e0243216. doi: 10.1371/journal.pone.0243216 (PMC7714190; doi:10.1371/journal.pone.0243216)
Supplement: S8 Table — (DOCX) [file pone.0243216.s009.docx]

**S8 Table.** Clinical characteristics of the non-drinkers and light drinkers with adjusted clinical status in the subjects who were not receiving drugs for hypertension, dyslipidemia and diabetes mellitus

| Variables | Alcohol consumption | | P value |
| --- | --- | --- | --- |
|  | None  0 g/week  (n=206) | Light  0 to <140 g/week  (n=206) |  |
| Age, years | 42±13 | 42±12 | 0.92 |
| Body mass index, kg/m^2^ | 20.8±2.8 | 20.8±2.8 | 0.99 |
| Systolic blood pressure, mm Hg | 116±17 | 113±16 | 0.07 |
| Diastolic blood pressure, mmHg | 73±11 | 71±12 | 0.05 |
| Heart rate, bpm | 65±9 | 62±8 | 0.002 |
| Total cholesterol, mg/dL | 192±38 | 195±33 | 0.37 |
| Triglycerides, mg/dL | 74±38 | 70±44 | 0.42 |
| HDL cholesterol, mg/dL | 67±14 | 73±15 | <0.001 |
| LDL cholesterol, mg/dL | 113±32 | 110±27 | 0.28 |
| γ-GTP, mg/dL | 18±13 | 23±26 | 0.03 |
| eGFR, mL/min/1.73m^2^ | 85.1±16.3 | 85.0±15.1 | 0.97 |
| Uric acid, mg/dL | 4.0±0.8 | 4.4±0.9 | <0.001 |
| Glucose, mg/dL | 90±16 | 90±9 | 0.69 |
| Hemoglobin A1c, % | 5.3±0.7 | 5.1±1.0 | 0.01 |
| Framingham risk score, % | 2.5±2.8 | 2.2±2.5 | 0.33 |
| Medical history, n (%) |  |  |  |
| Hypertension | 12 (5.8) | 12 (5.8) | 1.00 |
| Dyslipidemia | 40 (19.4) | 39 (18.9) | 0.90 |
| Diabetes mellitus | 2 (9.7) | 1 (4.9) | 0.56 |
| Hyperuricemia | 1 (4.9) | 2 (9.7) | 0.56 |
| Current smoker, n (%) | 0 (0) | 0 (0) | N/A |
| Flow-mediated vasodilation, % | 7.9±3.9 | 7.3±3.5 | 0.14 |

HDL indicates high-density lipoprotein; LDL, low-density lipoprotein; γ-GTP, gamma glutamyl transpeptidase; eGFR, estimated glomerular filtration rate; and N/A, not available.
