# Supplementary material for: Endothelial function is preserved in light to moderate alcohol drinkers but is impaired in heavy drinkers in women: Flow-mediated Dilation Japan (FMD-J) study
Source: PLoS One. 2020 Dec 3;15(12):e0243216. doi: 10.1371/journal.pone.0243216 (PMC7714190; doi:10.1371/journal.pone.0243216)
Supplement: S9 Table — (DOCX) [file pone.0243216.s010.docx]

**S9 Table**. Clinical characteristics of the non-drinkers and moderate drinkers with adjusted clinical status in the subjects who were not receiving drugs for hypertension, dyslipidemia and diabetes mellitus

| Variables | Alcohol consumption | | P value |
| --- | --- | --- | --- |
|  | None  0 g/week  (n=48) | Moderate  140< to 280 g/week  (n=48) |  |
| Age, yr | 38±11 | 38±11 | 1.00 |
| Body mass index, kg/m^2^ | 20.1±2.2 | 20.0±2.0 | 0.84 |
| Systolic blood pressure, mm Hg | 110±12 | 107±13 | 0.40 |
| Diastolic blood pressure, mmHg | 69±8 | 67±9 | 0.45 |
| Heart rate, bpm | 64±9 | 64±14 | 0.99 |
| Total cholesterol, mg/dL | 189±30 | 193±28 | 0.52 |
| Triglycerides, mg/dL | 63±26 | 66±38 | 0.65 |
| HDL cholesterol, mg/dL | 69±12 | 79±16 | <0.001 |
| LDL cholesterol, mg/dL | 110±28 | 102±25 | 0.15 |
| γ-GTP, mg/dL | 16±6 | 27±33 | 0.02 |
| eGFR, mL/min/1.73m^2^ | 89.2±14.2 | 88.9±16.6 | 0.93 |
| Uric acid, mg/dL | 4.1±1.0 | 4.2±1.0 | 0.35 |
| Glucose, mg/dL | 88±9 | 88±11 | 0.80 |
| Hemoglobin A1c, % | 5.3±0.3 | 5.3±0.3 | 0.43 |
| Framingham risk score, % | 1.6±1.6 | 1.4±0.9 | 0.39 |
| Medical history, n (%) |  |  |  |
| Hypertension | 0 (0) | 0 (0) | N/A |
| Dyslipidemia | 5 (10.4) | 5 (10.4) | 1.00 |
| Diabetes mellitus | 0 (0) | 0 (0) | N/A |
| Hyperuricemia | 0 (0) | 0 (0) | N/A |
| Current smoker, n (%) | 0 (0) | 1 (2.1) | 0.24 |
| Flow-mediated vasodilation, % | 8.5±2.6 | 8.5±4.1 | 0.98 |

HDL indicates high-density lipoprotein; LDL, low-density lipoprotein; γ-GTP, gamma glutamyl transpeptidase; eGFR, estimated glomerular filtration rate; and N/A, not available.
