# Supplementary material for: Endothelial function is preserved in light to moderate alcohol drinkers but is impaired in heavy drinkers in women: Flow-mediated Dilation Japan (FMD-J) study
Source: PLoS One. 2020 Dec 3;15(12):e0243216. doi: 10.1371/journal.pone.0243216 (PMC7714190; doi:10.1371/journal.pone.0243216)
Supplement: S10 Table — (DOCX) [file pone.0243216.s011.docx]

**S10 Table**. Clinical characteristics of the non-drinkers and heavy drinkers with adjusted clinical status in the subjects who were not receiving drugs for hypertension, dyslipidemia and diabetes mellitus

| Variables | Alcohol consumption | | P value |
| --- | --- | --- | --- |
|  | None  0 g/week  (n=20) | Heavy  >280 g/week  (n=20) |  |
| Age, yr | 38±13 | 38±13 | 1.00 |
| Body mass index, kg/m^2^ | 20.5±1.9 | 20.3±1.8 | 0.81 |
| Systolic blood pressure, mm Hg | 111±12 | 113±17 | 0.62 |
| Diastolic blood pressure, mmHg | 70±7 | 70±11 | 0.83 |
| Heart rate, bpm | 66±12 | 67±11 | 0.64 |
| Total cholesterol, mg/dL | 179±24 | 195±27 | 0.06 |
| Triglycerides, mg/dL | 60±25 | 72±54 | 0.37 |
| HDL cholesterol, mg/dL | 68±13 | 79±16 | 0.02 |
| LDL cholesterol, mg/dL | 101±23 | 100±26 | 0.94 |
| γ-GTP, mg/dL | 14±3 | 31±23 | 0.002 |
| eGFR, mL/min/1.73m^2^ | 87.3±17.1 | 84.5±15.4 | 0.59 |
| Uric acid, mg/dL | 4.1±0.7 | 4.8±1.4 | 0.049 |
| Glucose, mg/dL | 87±8 | 91±12 | 0.27 |
| Hemoglobin A1c, % | 5.3±0.2 | 5.0±1.1 | 0.31 |
| Framingham risk score, % | 1.7±1.3 | 2.5±2.9 | 0.27 |
| Medical history, n (%) |  |  |  |
| Hypertension | 1 (5.0) | 1 (5.0) | 1.00 |
| Dyslipidemia | 3 (15.0) | 3 (15.0) | 1.00 |
| Diabetes mellitus | 0 (0) | 0 (0) | N/A |
| Hyperuricemia | 0 (0) | 0 (0) | N/A |
| Current smoker, n (%) | 0 (0) | 1 (5.0) | 0.23 |
| Flow-mediated vasodilation, % | 8.8±3.3 | 6.5±1.8 | 0.007 |

HDL indicates high-density lipoprotein; LDL, low-density lipoprotein; γ-GTP, gamma glutamyl transpeptidase; eGFR, estimated glomerular filtration rate; and N/A, not available.
