# Supplementary material for: Endothelial function is preserved in light to moderate alcohol drinkers but is impaired in heavy drinkers in women: Flow-mediated Dilation Japan (FMD-J) study
Source: PLoS One. 2020 Dec 3;15(12):e0243216. doi: 10.1371/journal.pone.0243216 (PMC7714190; doi:10.1371/journal.pone.0243216)
Supplement: S1 File — (DOCX) [file pone.0243216.s012.docx]

1. 飲酒しますか？　（　はい　・　いいえ　）
2. 飲酒回数

（月1回　・　月２－３回　・　週１－２回　・　週３－４回　・　週５－６回　・　毎日　・　機会飲酒）

1. 種類

1. 量

1. Have you ever consumed alcohol? ( Yes ・ No )
2. How often do you have a drink containing alcohol?

( Once a month・2-3 times a month・1-2 times a week・3-4 times a week・5-6 times a week・every day・On special occasions)

1. What kind of drinks containing alcohol do you drink?

1. How much drinks containing alcohol do you have on a typical day?
